# Beta-blocker exposure and survival outcomes in patients with advanced pancreatic ductal adenocarcinoma: a retrospective cohort study (BETAPANC)

**DOI:** 10.3389/fphar.2023.1137791

**Published:** 2023-05-19

**Authors:** Antoine Le Bozec, Mathias Brugel, Zoubir Djerada, Marya Ayad, Marine Perrier, Claire Carlier, Damien Botsen, Pierre Nazeyrollas, Olivier Bouché, Florian Slimano

**Affiliations:** ^1^ CHU Reims, Oncology Day-Hospital, Reims, France; ^2^ CHU Reims, Service de Gastroentérologie et Oncologie Digestive, Reims, France; ^3^ Université de Reims Champagne-Ardenne, HERVI, Service Pharmacologie-Toxicologie, Reims, France; ^4^ Institut Jean Godinot, Département d’Oncologie Médicale, Reims, France; ^5^ Université de Reims Champagne-Ardenne, VieFra, CHU Reims, Service Cardiologie, Reims, France; ^6^ Université de Reims Champagne-Ardenne, Biospect, CHU Reims, Service de Gastroentérologie et Oncologie Digestive, Reims, France; ^7^ Université de Reims Champagne-Ardenne, Biospect, CHU Reims, Service Pharmacie, Reims, France

**Keywords:** adrenergic beta-antagonists, pancreatic neoplasms, cardiovascular diseases, drug repositioning, pharmacoepidemiogy

## Abstract

**Introduction:** Preclinical studies have demonstrated the possible role of beta-adrenergic receptors in pancreatic ductal adenocarcinoma (PDAC) tumor invasion and migration. The current study aimed to explore the possible association between survival outcomes and beta-blocker (BB) exposure in patients with advanced PDAC.

**Methods:** This retrospective single-center study included 182 patients with advanced PDAC. Clinical [age, sex, BMI, cardiovascular condition, presence (SBB) or absence (NSBB) of beta-1 selectivity of BB, exposure duration, and multimorbidity], oncological (stage and anticancer treatment regimen), and biological (renal and liver function) data were collected. The endpoints were overall survival (OS) and progression-free survival (PFS). Hazard ratios (HRs) and 95% confidence intervals (95% CIs) for survival outcomes associated with BB exposure were estimated using Cox regression model and propensity score (PS) methods.

**Results:** Forty-one patients (22.5%) were exposed to BB. A total of 104 patients progressed (57.1%) to PDAC and 139 (76.4%) patients died at the end of follow-up (median, 320 days; IQR, 438.75 days). When compared to the non-exposed group, there was no increase in survival outcomes associated with BB use (OS: HR = 1.38, 95% CI = 0.80–2.39, *p* = 0.25; PFS: adjusted HR = 0.95, 95% CI = 0.48–1.88, *p* = 0.88). Similar results were obtained using the PS method. Compared to no BB usage, SBB use was associated with a significant decrease in OS (HR = 1.80, 95% CI = 1.16–2.80, *p* < 10^−2^).

**Conclusion:** BB exposure was not associated with improved PDAC survival outcomes. Beta-1-selectivity was not independently associated with any differences.

## 1 Introduction

Pancreatic ductal adenocarcinoma (PDAC) is the fourth leading cause of cancer-related deaths in Europe (Rahib et al., 2014; Ferlay et al., 2016; Neuzillet et al., 2018), with a 5-year overall survival (OS) of 5%–8% (Cowppli-Bony et al., 2019). Given the poor prognosis and failure of innovative anticancer strategies (e.g., targeted therapies and immune checkpoint inhibitors), there is a need to look at the potential of drug repositioning or similar approaches identifying novel clinical use for an existing drug approved for non-oncological use (Pushpakom et al., 2019). Preclinical studies have suggested the involvement of beta-adrenergic pathways in the pathogenesis of various cancers (Lutgendorf et al., 2003; Sloan et al., 2010; Armaiz-Pena et al., 2013; Rains et al., 2017). Beta-adrenergic agonists of catecholamines (epinephrine and norepinephrine) have been shown to activate protein kinase A and mitogen-activated protein kinase (MAPK) pathways (Zhang et al., 2010; Cole and Sood, 2012). Activated transcription factors promote cell proliferation through high levels of catecholamines (stress and beta-adrenergic agonists). This mechanism has been shown to be activated in various preclinical cancer models (breast, colorectal, ovarian, lung, and pancreatic cancers) in favor of tumor growth and progression (Sloan et al., 2010; Al-Wadei et al., 2012; Rains et al., 2017).

It is now hypothesized that blockade of the beta-adrenergic pathway by adrenergic beta-antagonists, such as beta-blockers (BB), may have anticancer properties. Many retrospective studies have explored the impact of BB exposure on the overall (OS) and progression-free survival (PFS) in cohorts of melanoma, ovarian, breast, and lung cancer patients with conflicting results (Lemeshow et al., 2011; Aydiner et al., 2013; Watkins et al., 2015; Weberpals et al., 2017a; Jansen et al., 2017; Spera et al., 2017; Baek et al., 2018; Gillis et al., 2021). Some studies have also explored the impact of beta-1 selectivity on BB exposure, and Montoya et al. (2016) showed that the use of non-selective BB compared to beta-1 selective BB increased PFS in patients with early stage breast cancer. Two studies explored the impact of BB exposure on survival outcomes in PDAC with no significant benefit (Udumyan et al., 2017; Yang et al., 2021). Even though these studies used an interesting methodological approach, there was no specific characterization of cardiovascular conditions and the use of BB at diagnosis was considered an irreversible exposure. Because of the non-oncological primary purpose of BB, it is important to define cardiovascular conditions that could cause confusion bias among exposed and non-exposed patients when performing retrospective studies. Immortal time bias can occur when BB exposure is defined only at the start of follow-up, leading to a misclassified exposure for patients who had a BB prescription before or after the start of follow-up. By considering BB exposure as a time-dependent variable and using propensity score methods, it is possible to avoid inaccurate estimation of events associated with exposure (Weberpals et al., 2016; Weberpals et al., 2017b).

This BETAPANC study aimed to assess the effect of BB exposure on the OS and PFS of patients with advanced PDAC. Additionally, differences in survival outcomes between non-selective and selective BB were explored.

## 2 Material and methods

### 2.1 Study design and patients

This retrospective BETAPANC study was performed at a French tertiary hospital. Patients with advanced PDAC receiving intravenous anticancer treatment were screened from 23 November 2015, to 4 June 2022, from the Oncology day-hospital ONCOPTIMAL database that contains the best possible medication history, including all regular drugs such as BB, for all new patients. All data were collected from the ONCOPTIMAL database (registered in our tertiary hospital for personal data protection and in the Health Data Hub with reference number: F20220817142044). Because the ONCOPTIMAL database does not contain longitudinal information on survival outcomes, complementary studies using electronic health records (Easily, HCL, France) and the Computerized Physician Ordered Entry CHIMIO 5.9 (Computer Engineering, France) were used.

This study was conducted in compliance with the ethical principles of the Declaration of Helsinki. The database was built in accordance with the French law and the MR004 protocol (n°MR00425072022) of the *Commission Nationale de l’Informatique et des Libertés* (CNIL). Patient records were anonymized prior to analysis. As this study was non-interventional, retrospective, monocentric, and involved only electronic health record data collection, no informed consent or additional ethical committee review was required.

### 2.2 Procedures and data collection

Data collection included patient characteristics [age, sex, weight, and body mass index (BMI)], tumor characteristics (locally advanced or metastatic), and other morbidities with a focus on cardiovascular history that could be treated with BB [presence/absence of a diagnosis of arterial hypertension (AHT), myocardial ischemia (MI), cardiac arrhythmia (CA), or heart failure (HF)]. The presence of one of these pathologies describing the cardiovascular condition was screened in a specific cardiac consultation report or in multidisciplinary tumor board meetings. Data on anticancer chemotherapy-based regimen[a combination of 5-fluorouracil, leucovorin, irinotecan, and oxaliplatin (FOLFIRINOX), gemcitabine, or others] and biological data with a baseline before the first cycle of anticancer therapy [serum glutamate pyruvate transaminase (SGPT), serum glutamic oxaloacetate transferase (SGOT), total bilirubin, serum albumin, alkaline phosphatase, serum potassium, and serum creatinine] were also collected. Best Possible Medication History (BPMH) allowed for the collection of data on each drug and its dosage. BB exposure is described as follows: Drug name, indication, and beta-1 selectivity. Multimorbidity was defined as at least five comorbidities (excluding PDAC), and polypharmacy was defined as the presence of at least five prescribed drugs.

### 2.3 Outcomes and endpoints

Survival outcomes in patients with advanced PDAC were analyzed and compared between the following groups: BB exposure (BB+), BB non-exposure (BB-), non-selective BB exposure (NSBB+), and selective BB exposure (SBB+). OS was defined as the time from inclusion (administration of first anticancer treatment) to all-cause death or the last follow-up. PFS was defined by progression confirmed from clinical and radiological assessment registered as such in the electronic health record. The primary endpoint was OS in patients with advanced PDAC between BB+ and BB-. The secondary endpoints were PFS for patients with advanced PDAC between BB+ and BB-, and OS and PFS for patients with advanced PDAC (BB+) between NSBB+ and SBB+.

### 2.4 Statistical analysis

Quantitative variables were described as mean ± standard deviation (SD) or median (interquartile range, minimum, and maximum) according to the distribution of the variable, and qualitative variables were described as numbers and percentages. Student’s *t*-test was used to compare the means, and Fischer’s exact test was used to compare the percentages between BB exposure and non-exposure. Univariate and multivariate Cox proportional hazards regression models were used to estimate the association between BB ex posure as well as the endpoints of OS and PFS occurrence using the likelihood ratio test to determine *p*-values. The proportional hazard assumption was verified for qualitative and quantitative variables using the Therneau test and Schoenfeld residuals. The log-linearity assumption was verified for the quantitative variables using Martingale residuals. Exposure variables were considered as dichotomous time-dependent variables to limit the effect of immortal time bias by collecting exposure status at the inclusion and last date of follow-up (death, progression, or end of study inclusion period). Multivariate logistic regressions were achieved with a stepwise backward procedure. In addition, to help account for the non-randomized treatment administration of BB exposure, propensity score (PS) methods were used to reduce the effects of confounding bias (Supplementary Material). PS is defined as the probability of treatment assignment conditional on the observed baseline characteristics. As explained by Brookhart et al., variables included in the multivariate logistic regression to calculate the PS should be associated with the occurrence of the event and treatment attribution (Brookhart et al., 2006). A Cox proportional hazards model was designed using three PS methods: PS as an additional covariate, PS matching, and inverse probability of treatment weighting (Austin, 2007; Austin, 2011; Forbes and Shortreed, 2008; Abadie and Imbens, 2009; Xu et al., 2010; Connolly and Gagne, 2016). The estimated propensity score was directly included in a multivariate model, comprising two explanatory variables of the endpoint: exposure (BB treatment) and propensity score. The results were presented as hazard ratios (HRs) and 95% confidence intervals (CIs). PS discrimination was validated by determination of the Receiver Operating Characteristic (ROC) curve (Supplementary Material). Because of the potential for type I error due to multiple comparisons, findings for the analyses of primary and secondary endpoints should be interpreted as exploratory. All analyses were performed using the R software^®^ (v4.1.0).

## 3 Results

### 3.1 Baseline patient characteristics

**FIGURE 1:**
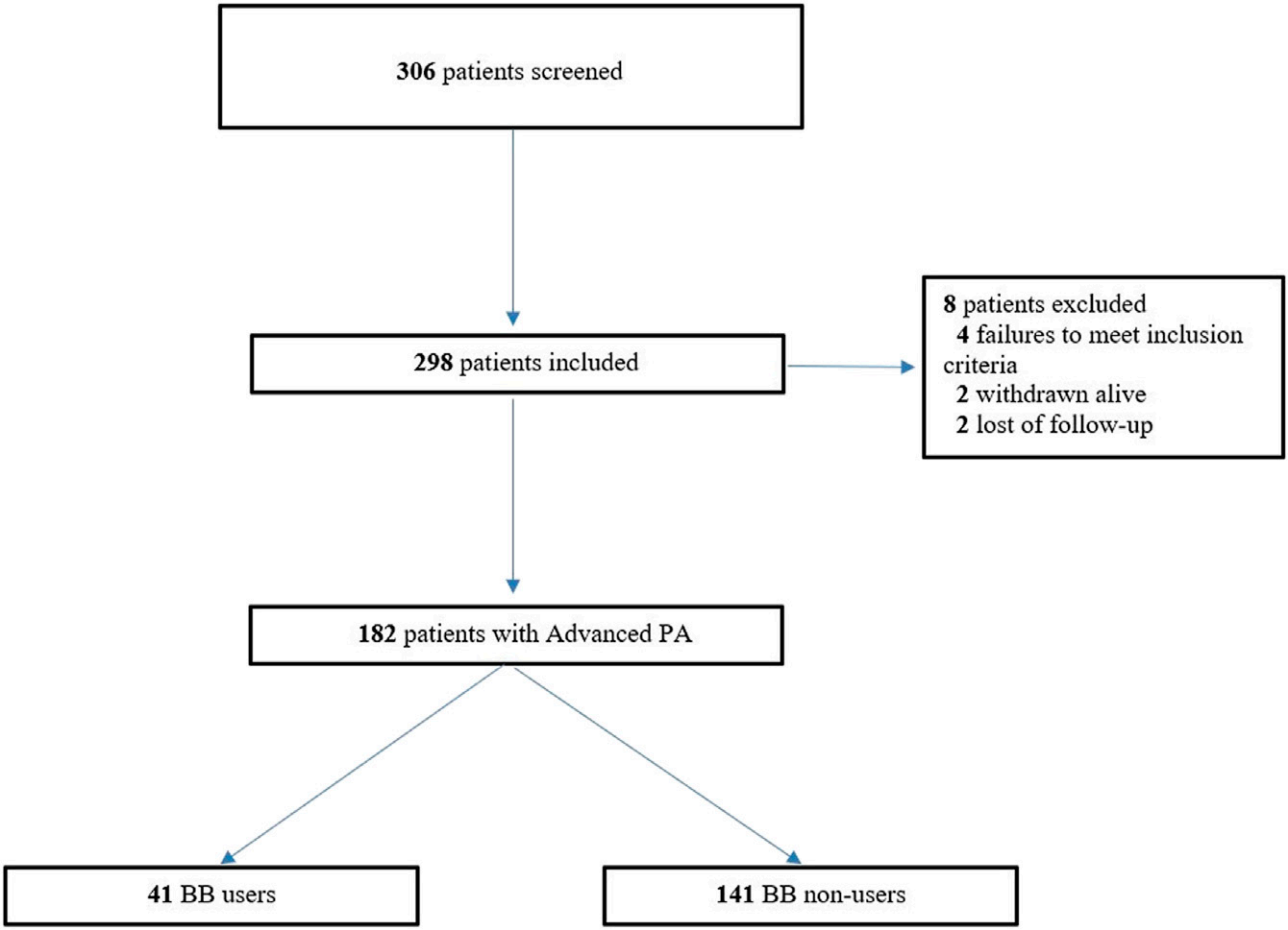
Flow Chart of the cohort study of patients with PDAC.

The inclusion criteria were 298 eligible patients with PDAC from the ONCOPTIMAL database (Figure 1; Table 1). Among the included patients, 182 (61.1%) had advanced PDAC; 41/182 (22.5%) had BB+. The BB exposure (70.7% vs. 50.4%; *p* = 0.03) was higher in males, patients with higher weight and lower eGFR (categorical) (*p* < 0.05) (Peixoto et al., 2015). We also observed multimorbidity (BB+:5.15 ± 2.76 comorbidities vs. BB-:3.49 ± 2.59 comorbidities; *p* < 10^−2^), cardiovascular condition (except for HF), and polypharmacy (BB+:9.20 ± 4.64 drugs vs. BB-:6.52 ± 4.23 drugs; *p* < 10–2) in BB users. Furthermore, we found comparable distributions of anticancer regimen with fluorouracil in both exposition groups [BB+: 32/41 (78%) vs. BB-: 110/141 (78.0%)].

**TABLE 1:** Baseline patient’s characteristics according to beta-blocker exposure.

Characteristics	Total (*n* = 182)	BB users (*n* = 41)	BB non-users (*n* = 141)	*p*-value
Age (continuous) (m ± Standard Deviation)	65.73 ± 10.27	68.04 ± 9.95	65.06 ± 10.30	0.10
Age (categorial) n (%)				
18–59 years	51 (28.0)	6 (14.6)	45 (31.9)	0.10
60–69 years	54 (29.7)	14 (34.1)	40 (28.4)	
>70 years	77 (42.3)	21 (51.3)	56 (39.7)	
Sex n (%)				
Male	100 (54.9)	29 (70.7)	71 (50.4)	0.03
Female	82 (45.1)	12 (29.3)	70 (49.6)	
Weight kg (m ± SD)	69.66 ± 15.58	74.12 ± 15.15	68.36 ± 15.51	0.04
BMI kg/m^2^ (continuous) (m ± SD)	24.37 ± 4.41	25.39 ± 3.59	24.09 ± 4.59	0.06
*NA*	1	1	0	
BMI kg/m^2^ (categorial)				
18–25 kg/m^2^	104 (57.1)	18 (43.9)	86 (61.0)	0.13
25–30 kg/m^2^	58 (31.9)	18 (43.9)	40 (28.4)	
>30 kg/m^2^	19 (10.4)	4 (9.8)	15 (10.6)	
*NA*	1 (0.6)	1 (2.4)	0	
Serum creatinine value µmol/L (m ± SD)	67.36 ± 21.60	73.65 ± 19.50	65.50 ± 21.90	0.03
*NA*	3	0	3	
eGFR (categorial) n (%)				
Stade I (≥90 mL/min/1.73m^2^)	140 (76.9)	27 (65.8)	113 (80.1)	0.05
Stades II-IIIa (<90 mL/min/1.73m^2^)	39 (21.4)	14 (34.2)	25 (17.7)	
NA	3 (1.7)	-	3 (2.2)	
Potassium level (m ± SD)	4.56 ± 0.52	4.62 ± 0.52	4.54 ± 0.53	0.63
*NA*	133	30	103	
SGPT (m ± SD)	43.80 ± 45.11	56.48 ± 73.88	40.12 ± 31.88	0.20
*NA*	13	3	10	
SGOT (m ± SD)	55.98 ± 59.61	57.79 ± 62.87	55.54 ± 69.87	0.81
*NA*	13	3	10	
Total bilirubinemia (µmol/L)	18.44 ± 29.08	25.25 ± 43.40	16.44 ± 23.14	0.22
*NA*	8	2	6	
Serum albumin (g/L)	39.15 ± 5.70	38.43 ± 4.02	39.37 ± 6.13	0.31
*NA*	43	8	35	
ALK (UI/L)	241.75 ± 282.41	270.76 ± 405.66	233.10 ± 235.06	0.59
*NA*	21	4	17	
Anticancer regimen 1				
FOLFIRINOX	105 (57.7)	24 (58.5)	81 (57.4)	0.90
Gemcitabine	37 (20.3)	9 (22.0)	28 (19.9)	
Other capecitabin-oxaliplatin, LV5FU2, FOLFIRI, FOLFOX, FOLFOX-bevacizumab	40 (22.0)	8 (19.5)	32 (22.7)	
Anticancer regimen 2				
Chemotherapy with fluorouracil	142 (78.0)	32 (78.0)	110 (78.0)	0.99
Chemotherapy with no fluorouracil	40 (22.0)	9 (22.0)	31 (22.0)	
Comorbidities				
Global (m ± SD)	3.86 ± 2.71	5.15 ± 2.76	3.49 ± 2.59	<10^−2^
Categories n (%)				
*<5*	120 (65.9)	18 (43.9)	102 (72.3)	<10^−2^
*≥5*	62 (34.1)	23 (56.1)	39 (27.7)	
Cardiovascular condition n (%)	***n* = 92 (50.5)**	*n* = 40	*n* = 52	
AHT	79 (43.4)	29 (70.7)	50 (35.5)	<10^−3^
CA	13 (7.1)	10 (24.4)	3 (2.1)	<10^−5^
HF	2 (2.2)	0	2 (1.3)	0.99
MI	20 (11.0)	13 (31.7)	7 (5.0)	<10^−5^
Cumulated cardiovascular comorbidities n (%)				
1	72 (39.6)	28 (68.3)	44 (31.2)	<10^−10^
≥2	22 (12.1)	13 (31.7)	9 (6.4)	
Polypharmacy				
Global (m ± SD)	7.12 ± 4.46	9.20 ± 4.64	6.52 ± 4.23	<10^−2^
Categorial n (%)				
<5	57 (31.3)	7 (17.0)	50 (35.5)	0.01
Polypharmacy (5–9)	78 (42.9)	17 (41.5)	61 (43.3)	
Excessive Polypharmacy (≥10	47 (25.8)	17 (41.5)	30 (21.3)	

BB, beta-Blocker; BMI, body mass index; eGFR, estimated Glomerular Filtration Rate; SGPT, serum glutamate pyruvate transaminase; SGOT, Serum Glutamo-oxaloacetate Transferase; ALK, alkaline phosphatase; FOLFIRINOX, irinotecan, oxaliplatin, leucovorin, 5-FU; LV5FU2, leucovorin, 5-FU; FOLFIRI, irinotecan, leucovorin, 5-FU; FOLFOX, leucovorin, 5-FU, oxaliplatin; AHT, arterial hypertension; CA: cardiac arrythmia; HF: heart failure; MI: myocardial ischemia.

Bold and italic values correspond to the number of patients with at least one cardiovascular condition, but could have more than one cardiovascular condition.

### 3.2 Primary outcome

**FIGURE 2:**
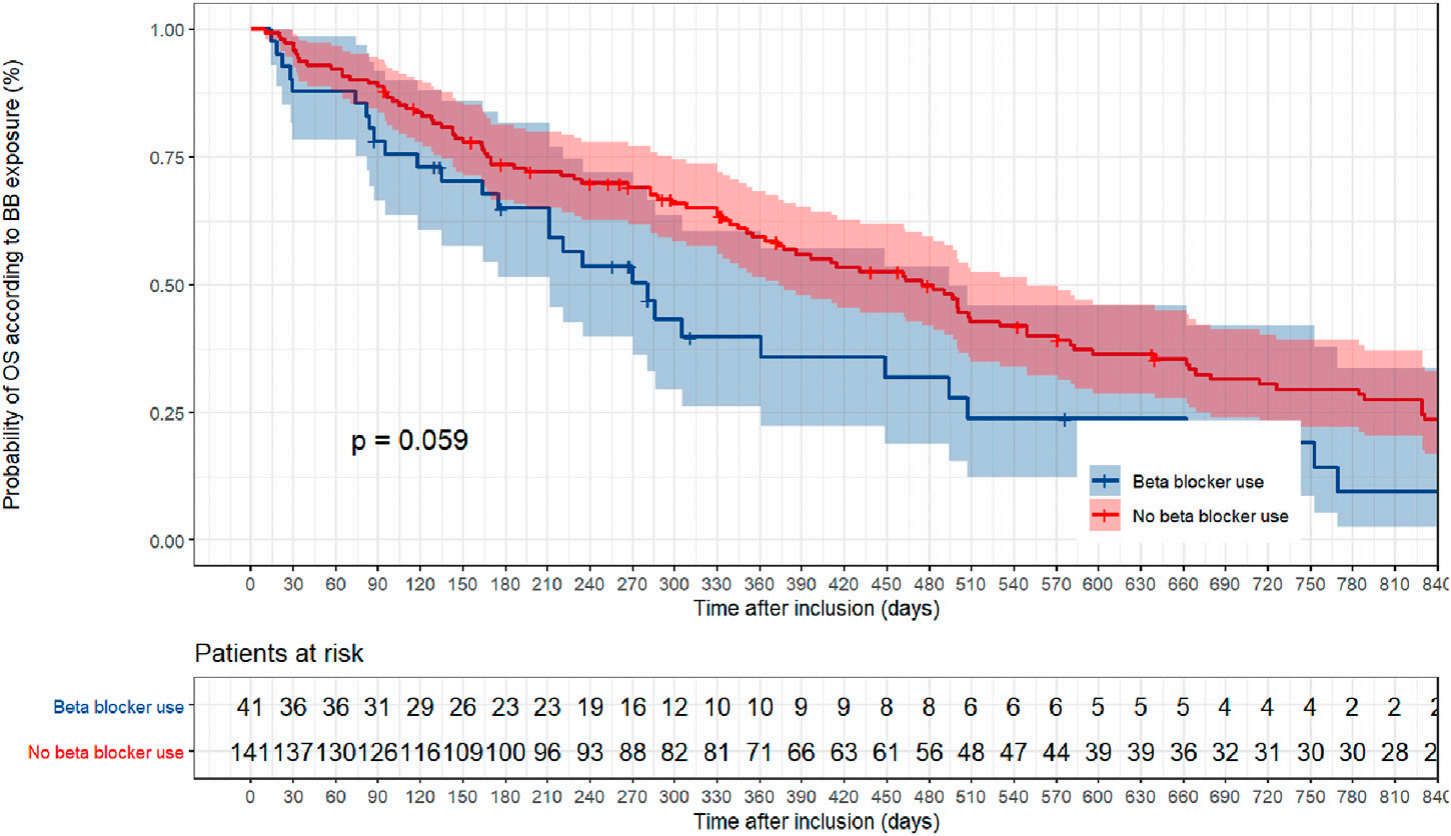
Non parametric Kaplan–Meier estimation of overall survival based on beta-blocker exposure.

Death occurred in 30 of 41 BB + patients (73.2%) and 109 of 141 BB- patients (77.3%). The median OS was 281 and 475 days for BB+ and BB- patients, respectively. In BB + patients, the log-rank test showed a non-significant increase in the risk of death (*p* = 0.06) (Figure 2). BB + patients had a higher but non-significant risk of death (HR = 1.38; 95% CI = 0.80–2.39) according to the Cox regression multivariate analysis adjusted for age, sex, eGFR, anticancer regimen, polypharmacy, multimorbidity, cardiovascular condition, and use of BB (AHT, MI, CA, but no HF). When considering BB exposure as a time-dependent variable, there was a non-significant effect compared to BB exposure as a non-time-dependent variable (HR = 1.02, 95% CI, 0.10–1.45; *p* = 0.98). Similar results were found with PS adjustment (HR = 1.28, 95% CI, 0.82–2.00; *p* = 0.27), PS matching (HR = 1.45, 95% CI, 0.81–2.63; *p* = 0.22) and inverse probability of treatment weighting (IPTW) (HR = 1.31, 95% CI, 0.83–2.09; *p* = 0.25) all three showed a non-significant increase in death risk (Table 2; Supplementary Figures S1–S3). The results of the univariate and multivariate coefficients are shown in Supplementary Tables S1, S2.

**TABLE 2:** Associations between beta-blocker exposure and death in the crude analysis, multivariable analysis, and propensity score analyses.

Parameters	Results	*p*-value**
Number of events/number of patients at risk (%)		
BB + patients (*n* = 41)	30 (73.2%)	—
BB- patients (*n* = 141)	109 (77.3%)	—
Crude analysis–HR (95% CI) *	1.48 [0.98–2.23]	0.06
**Multivariable analysis** ^**‡**^	1.38 [0.80–2.39]	0.25
**PS analysis**		
Adjusted for PS^§^	1.28 [0.82–2.00]	0.27
With matching^†^	1.45 [0.81–2.63]	0.22
With inverse probability-weighting^φ^	1.31 [0.83–2.09]	0.25

BB, beta-blocker; HR: hazard ratio; CI, confidence interval; PS, propensity score.

*All variables integrated in Cox regression models validated proportional hazard and log-linearity assumptions (for continuous variables).

**Likelihood ratio test.

‡Hazard ratio from the multivariable Cox proportional-hazards model, with adjustment for age, gender, anticancer regimen, multimorbidity, polypharmacy, presence of cardiovascular comorbidity, AHT, MI, CA. The analysis included 182 patients.

§Hazard ratio from a multivariable Cox proportional-hazards model with additional adjustment for the propensity score. The analysis included 182 patients.

†Hazard ratio from a multivariable Cox proportional-hazards model with matching according to the propensity score. The analysis included patients 60 patients (30 who received BB, and 30 who did not) according to the nearest method with a caliper fixed at 0.2.

φ Hazard ratio from the multivariate Cox proportional-hazards model with inverse probability weighting according to the propensity score. The analyses included 182 patients.

### 3.3 Secondary outcomes

#### 3.3.1 Determination of PFS according to BB exposure

**FIGURE 3:**
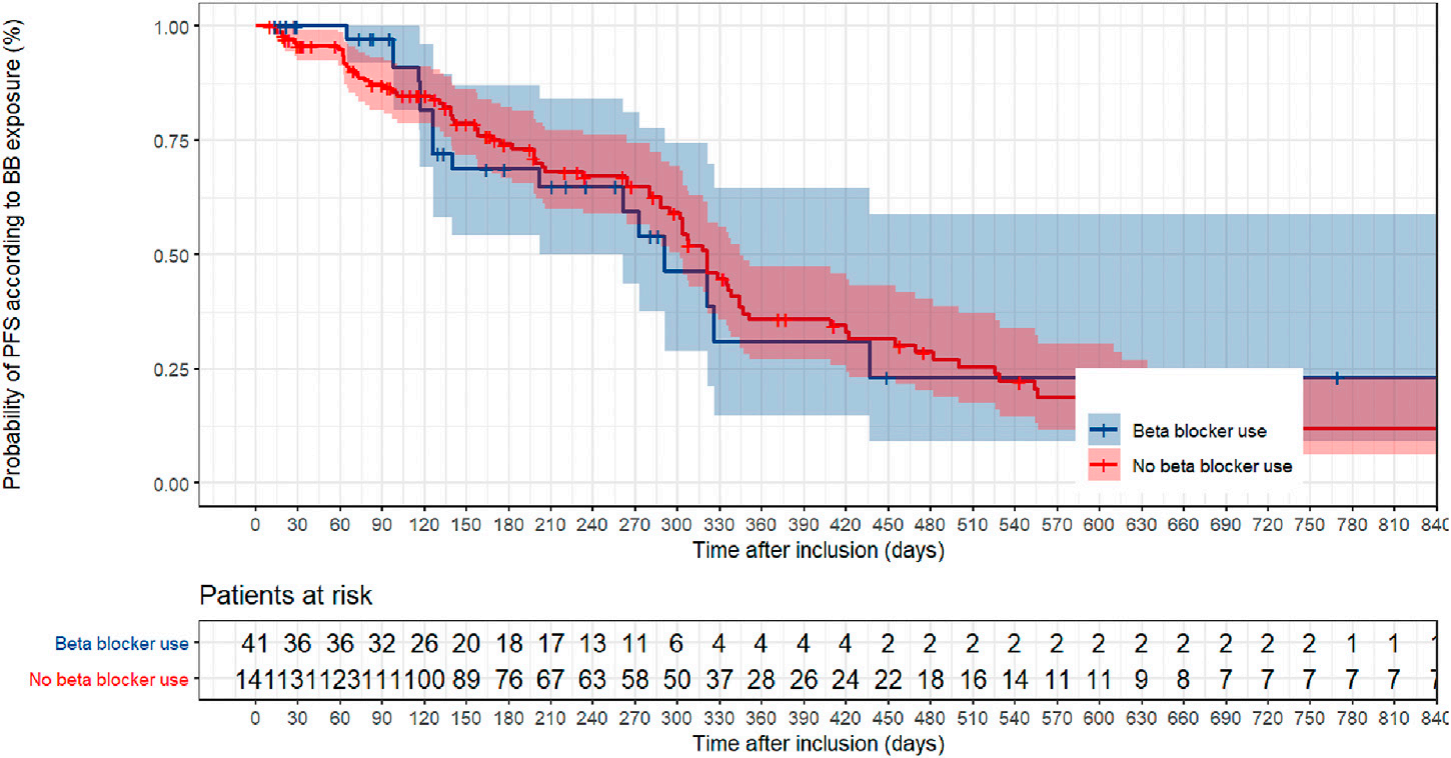
Non parametric Kaplan–Meier estimation of progression-free survival based on beta-blocker exposure.

Progression occurred in 18 of 41 BB + patients (43.9%) and 86 of 141 BB- patients (61.0%). The median PFS was 291 and 321 days for BB+ and BB-, respectively. In BB + patients, the log-rank test showed a non-significant increase in the risk of progression (*p* = 0.99) (Figure 3). BB + patients had a non-significant decrease in progression risk (HR = 0.95; 95% CI = 0.48–1.88) according to the Cox regression multivariate analysis adjusted for multimorbidity, anticancer regimen, cardiovascular condition, and AHT. In the time-dependent analysis, a non-significant decrease in the progression risk was found when considering BB exposure as a time-dependent variable compared to BB exposure as a non-time-dependent variable (HR = 0.27, 95% CI, 0.065–1.12; *p* = 0.07). Similar results were found with PS methods: non-significant effect using PS adjustment (hazard ratio, HR = 1.01, 95% CI, 0.51–2.01; *p* = 0.97), non-significant decrease in the risk of progression with (HR = 0.73, 95% CI, 0.34–1.56; *p* = 0.42) and inverse probability of treatment weighting (IPTW) (HR = 0.96, 95% CI, 0.54–1.70; *p* = 0.90). (Supplementary Tables S3–S5).

#### 3.3.2 Determination of OS according to BB selectivity

**FIGURE 4:**
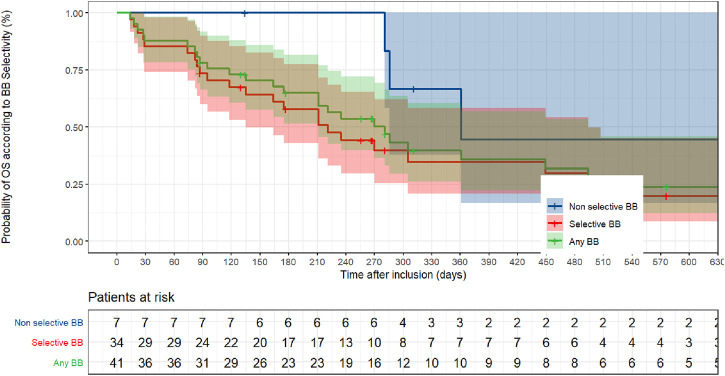
Non parametric Kaplan–Meier estimation of overall survival based on beta-blocker selectivity.

Among the 41 BB + patients, 34 were SBB+ and seven were NSBB+. The risk of death was not significantly reduced in NSBB + patients. Moreover, it significantly increased in SBB + patients (HR = 1.80, 95% CI, 1.16–2.80; *p* = 0.009) (Figure 4; Table 3).

**TABLE 3:** Overall Survival according to betablocker selectivity (versus beta-blocker non-users).

Parameters	Nb of events (%)	Univariate analysis	*p*-value
Selectivity		Ref = No beta-blocker	
*NSBB+ (n = 7)*	5 (71.4)	HR = 0.77 [0.31–1.94]	0.58
*SBB+ (n = 34)*	25 (73.5)	HR = 1.80 [1.16–2.80]	<10^−2^

NSBB+, Non Selective beta-blocker Use; SBB+, Selective beta-blocker Use.

## 4 Discussion

In the present study, BB exposure was not associated with an improvement in OS or PFS outcomes in patients with advanced PDAC. The results were similar when BB exposure was considered as a time-dependent variable and the PS method was used. There was a significant decrease in OS in patients with SBB + advanced PDAC.

These results contradict the first clinical report from a Swedish cohort study including 2,394 patients with all types of PDAC, which showed a lower mortality risk in patients with BB administration (HR, 0.79; 95% CI, 0.70–0.90; *p* < 0.001). This rate reduction was higher in patients with localized PDAC at diagnosis (HR, 0.60; 95% CI, 0.43–0.83; *p* = 0.002), who were excluded from our inclusion criteria (Udumyan et al., 2017). Although the Swedish study considered a pre-exposure delay of 90 days before inclusion, the present study defined exposition only at inclusion. These contradictory results could be explained by considering exposure as a time-dependent variable, and we used the PS method to describe this exposure. Moreover, the Swedish study period was from 2006 to 2009, and advances in PDAC management have been substantial since 2009, especially with the introduction of the FOLFIRINOX regimen (Conroy et al., 2011). Two other studies have explored the hypothesized benefit of BB in patients with advanced PDAC. Udumyan et al. (2017) found a significant decrease in cancer-specific mortality (adjusted HR, 0.79; 95% CI, 0.70–0.90; *p* < 0.001), and even more in patients with localized disease at diagnosis (adjusted HR, 0.60; 95% CI, 0.43–0.83; *p* = 0.002), with no significant differences in survival according to BB selectivity. To allow comparison with these results, in addition to advanced PDAC, survival in patients with localized disease should be explored. Furthermore, it should be noted that survival results were not adjusted for cardiovascular conditions. In the second study, Yang et al. (2021) found no significant survival differences according to BB exposure after stratification by conditions with indications for BB exposure. We showed comparable results, except for patients with CA who had a significant decrease in PFS.

To counterbalance the non-inclusion of BB exposure before the commencement of anticancer treatment and to assume that exposure status was not necessarily the same during the entire follow-up, a time-dependent BB exposure variable was included in the Cox regression models. This dichotomous variable ensured that we did not underestimate event occurrence risk. We found no significant difference when using BB treatment as a time-dependent variable, even though we observed a non-significant protective effect of BB exposure on progression probability. Nevertheless, time-dependent analyses should be interpreted cautiously and should be confirmed in prospective studies with larger sample sizes. This method was used in a previous study by Johannesdottir et al. (2013) to better capture the effect of BB exposure on OS in a cohort of patients with ovarian cancer. Udumyan et al. (2017) performed an intention-to-treat analysis approach (ITT analysis) by considering that exposure defined 90 days before could not evolve during follow-up.

Immortal time bias is an important limitation of retrospective studies that focus on survival outcomes (Weberpals et al., 2017b). Pharmacoepidemiological studies in patients with PDAC are complex because of the poor prognosis of the disease, leading to potential confounding bias. In our study, we selected all eligible patients to receive anticancer treatment even with frailty (older age and multimorbidity). There were significant differences between the groups; however, Cox proportional hazards modeling was performed with regression adjustment for these confounders, including cardiovascular conditions. It is well known that BB is a valuable drug for managing cardiovascular conditions that have moderate and high morbidity potential, and the study design must clearly dissociate death risk related to BB exposure and cardiovascular conditions. Baek et al. (2018) analyzed the impact of BB exposure on ovarian cancer, taking cardiovascular conditions into account. The present study added to the multivariate analyses with different variables supposed to capture cardiovascular bias: presence or absence of at least one cardiovascular condition, each cardiovascular condition (AHT, MI, and CA, excluding HF), and cumulative cardiovascular condition. The use of PS methods in the analysis reduces the risk of non-comparability. The calculation of the PS was performed using a logistic regression based on pre-existing studies that had already used PS to analyze BB exposure in cohorts of patients with ovarian, breast, lung, and colorectal cancer (Johannesdottir et al., 2013; Cardwell et al., 2016; Weberpals et al., 2017a; Jansen et al., 2017). Similar results were observed in the present study using the same methods.

This study aimed to assess the effect of BB exposure on BB beta-1 selectivity. Even though our subgroup sizes were small, we found a higher risk of death in SBB + patients than in NSBB + patients. Similar results have been reported in a retrospective study of 404 breast cancer patients, with a significant benefit in patients with NSBB on PFS (Montoya et al., 2016). In our study, most patients in the BB + group received SBB, whereas beta-2 receptors seemed to be more involved in carcinogenesis, such as cancer cell proliferation, angiogenesis, and cell migration (Zhang et al., 2010; Montoya et al., 2016). The greater survival outcomes with NSBB could be explained by preclinical evidence. Several preclinical studies have explored the effects of beta-adrenergic receptor blockade in human pancreatic carcinoma cell lines (PC-2). Guo *et al.* reported the predominant expression of beta-2-AR compared to beta-1-AR in the human pancreatic cancer cell lines Miapaca-2 and Bxpc-3 and observed that the neuroendocrine system increased the expression of MMP-2, MMP-9, and VEGF-A due to neuroendocrine activation. These effects were inhibited by the NSBB propranolol (Guo et al., 2009). Furthermore, Zhang et al. (2009) compared the rate of increase in the number of apoptotic PC-2 cells according to the type of BB, and found it to be lowest with SBB metoprolol. The proposed hypotheses were that beta-2 adrenergic antagonists suppressed invasion and proliferation by inhibiting both cAMP/PKA and Ras pathways, which regulate the activation of the MAPK pathway and transcription factors, such as NF-κB, AP-1, and CREB, as well as the expression of its target genes, *MMP-9*, *MMP-2*, and *VEGF*. However, beta-1 adrenergic antagonists suppress cell invasion by inhibiting the cAMP/PKA pathway (Zhang et al., 2010). It is also important to note that BB + patients were more frequently male and older than BB- patients, which could explain why BB + patients were more exposed to cardiovascular conditions and had a higher risk of death. Furthermore, considering these pre-existing conditions and the poor prognosis of PDAC, it is difficult to properly assess the impact of BB exposure in these cancer patients. Shah *et al.* found similar poorer survival results in pancreatic cancer patients using BB compared to other localized cancers localized, such as lung, breast, or colorectal cancer, thus confirming that the degree of severity of PDAC could be a real strength in studies exploring the impact of repurposing drugs such as BB (Sm et al., 2011). Limited by the number of available articles as well as because of the heterogeneity due do primary type of cancer from studies that explored the effect of BB on anticancer-treatment outcomes, it seems difficult to estimate the number of event needed to correctly assess statistical power (see Supplementary Methods). Notably, immune checkpoint inhibitors are used for many cancer localizations but not for PDAC. Knowing the contribution of beta-AR signaling to the increasing efficacy of immunotherapy could explain the better survival observed in immunogenic cancers such as melanoma, lung, and breast cancer (Kokolus et al., 2018; Mellgard et al., 2022).

Finally, although no association between BB exposure and survival outcomes was observed, there was no specific detailed characterization of cardiovascular conditions in any of these studies that could cause confusion bias among exposed and non-exposed populations. We have not considered specific causes of death due to cancer by using, for example, competing risk models such as Fine and Gray analysis, and the cause of death was mainly attributed to cancer or its progression.

The present study had other limitations. First, we could only collect information at the time of inclusion, and we could not identify risk of event occurrence according to the history of delayed prescription. In addition, the cumulative dose effect was not collected. Spera et al. (2017) showed an increase in PFS when BB exposure occurred after diagnosis compared to exposure before diagnosis in patients with advanced HER2-negative breast cancer. Furthermore, because BB is a regular drug that is administered by the patients themselves without daily control, potential measure bias could occur due to poor adherence to guidelines. Other prognostic factors were not integrated in our study, such as smoking history, CA 19–9 levels, Type 1 human ether-a-go-go-related gene (hERG1) and monitoring biologic values such as bilirubin, serum albumin, and alkaline phosphatase levels, because of missing data (Maisey et al., 2005; Lastraioli et al., 2015a; Lastraioli et al., 2015b; Arcangeli et al., 2022; Prognostic factors and sites of metastasis in unresectable locally advanced pancreatic cancer - PubMed, n. d.).

## 5 Conclusion

The present study did not show any significant benefit of BB exposure on the survival outcomes of patients with advanced PDAC. Similar results were obtained using PS methods and considering exposure as a time-dependent variable. Better control of BB exposure by running larger and interventional studies could provide a better understanding of the hypothetical potential of BB in patients with advanced PDAC and minimize any methodological bias as best as possible.

## Data Availability

The original contributions presented in the study are included in the article/Supplementary Material, further inquiries can be directed to the corresponding author.
