# Supplementary material for: Beta-blocker exposure and survival outcomes in patients with advanced pancreatic ductal adenocarcinoma: a retrospective cohort study (BETAPANC)
Source: Front Pharmacol. 2023 May 19;14:1137791. doi: 10.3389/fphar.2023.1137791 (PMC10235451; doi:10.3389/fphar.2023.1137791)
Supplement: Supplementary file 1 [file DataSheet1.docx]

Supplementary Material

**TITLE PAGE**

**Type of manuscript: Original article**

**Beta-blocker exposure and Survival Outcomes in Patients with advanced Pancreatic Ductal Adenocarcinoma: a retrospective cohort study (BETAPANC)**

Short title: Beta-blocker use and survival in advanced pancreatic cancer patients

Antoine Le Bozec^1*^, Mathias Brugel^2†^, Zoubir Djerada^3†^, Marya Ayad^1^, Marine Perrier^4^, Claire Carlier^1,5^, Damien Botsen^4,5^, Pierre Nazeyrollas^6^, Olivier Bouché^7†^, Florian Slimano^8†^

^1^ CHU Reims, Oncology Day-Hospital, F-51100 Reims, France

^2^ CHU Reims, Service de Gastroentérologie et oncologie digestive, F-51100 Reims, France

^3^ Université de Reims Champagne-Ardenne, HERVI, Service Pharmacologie-Toxicologie, F-51100 Reims, France

^4^ CHU Reims, Service de Gastroentérologie et oncologie digestive, F-51100 Reims, France

^5^ Institut Jean Godinot, Département d’oncologie médicale, F-51100 Reims, France

^6^ Université de Reims Champagne-Ardenne, VieFra, CHU Reims, Service Cardiologie, F-51100 Reims, France

^7^ Université de Reims Champagne-Ardenne, BioSpecT, CHU Reims, Service de Gastroentérologie et oncologie digestive, F-51100 Reims, France

^8^ Université de Reims Champagne-Ardenne, BioSpecT, CHU Reims, Service Pharmacie, F-51100 Reims, France

***Corresponding Author:**

Dr. Antoine Le Bozec, Pharm.D., M.Sc.

Oncology Day-Hospital (UMA-CH), CHU Reims, Rue Général Koenig, 51100 Reims, France

Phone: +33 0326787373 ; Fax: +33 0326787800 ; antoinelbz75@gmail.com

† These authors contributed equally

# Supplementary Methods

## Number of needed events

## “We used three different references source (related to the topic) for the determination of the needed events size:

## In the one prospective study (as we know), De Giorgi et al. found a lower occurrence progression and death in cohort of melanoma patients in the betablocker group (propranonol) with Hazard ratios (HR) (for PFS, HR = 0.018 [0.04-0.89], pval=0.03; for OS, HR = 0.64 [0.10-3.96], pval=0.63), respectively (De Giorgi et al., 2018). Authors reported 10.5% patients with death occurrence in BB+ group and 17.7% in BB- group. They also reported 15.8% patients with progression occurrence in BB+ group and 41.2% in BB- group. Finally, those proportions were used to determine the number of events required through nQuery® software: 191 events were needed in our study with an alpha risk of 5% and a power of 80%.

## This first estimation could be biased by the large difference in median follow up between cohort of melanoma patients in the study of De Giorgi et al. and cohort of advanced pancreatic cancer patients. We used results from a retrospective study that investigate death occurrence in pancreatic cancer patients cohort according to BB exposure (Udumyan et al., 2017; Yang et al., 2021). The number of events required through nQuery® software were 684 events were needed in our study with an alpha risk of 5% and a power of 80%.”

## Variables integrated in Cox regression models

Variables integrated in the Cox regression multivariate models differed according to the endpoint:

1. For the primary endpoint (OS), variables selected in multivariate analysis were: Age, Sex, Anticancer treatment regimen, polypharmacy status, multimorbidity, presence of cardiovascular condition, Arterial Hypertension (AHT), Myocardial Ischemia (MI), Cardiac Arrhythmia (CA), or Heart failure (HF).
2. For the secondary endpoint (PFS) variables selected in multivariate analysis were: Anticancer treatment regimen, multimorbidity, cumulated cardiovascular conditions and Arterial Hypertension.

Variables could differ between primary and secondary endpoint because of the use of “Backward stepwise method” as selection method of variables in our multivariate models. This method allowed us to start with a model that contains all variables under consideration then we removed the least significant variables one after the other. The least significant variable is defined by:

- the highest p-value in the model, or
- Its elimination from the model causes the lowest drop in R2, or
- Its elimination from the model causes the lowest increase in RSS (Residuals Sum of Squares) compared to other predictors

# Supplementary Figures and Tables

## Supplementary Figures


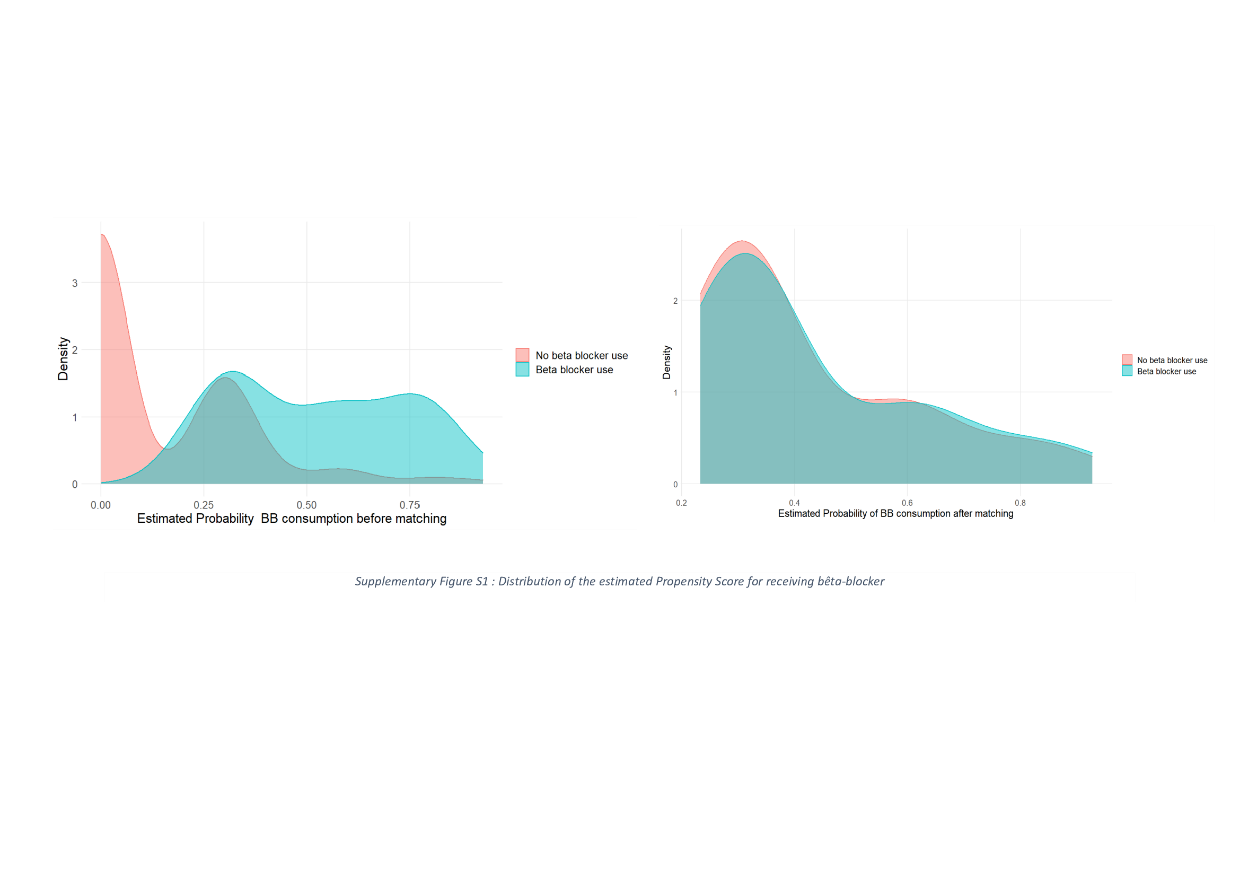


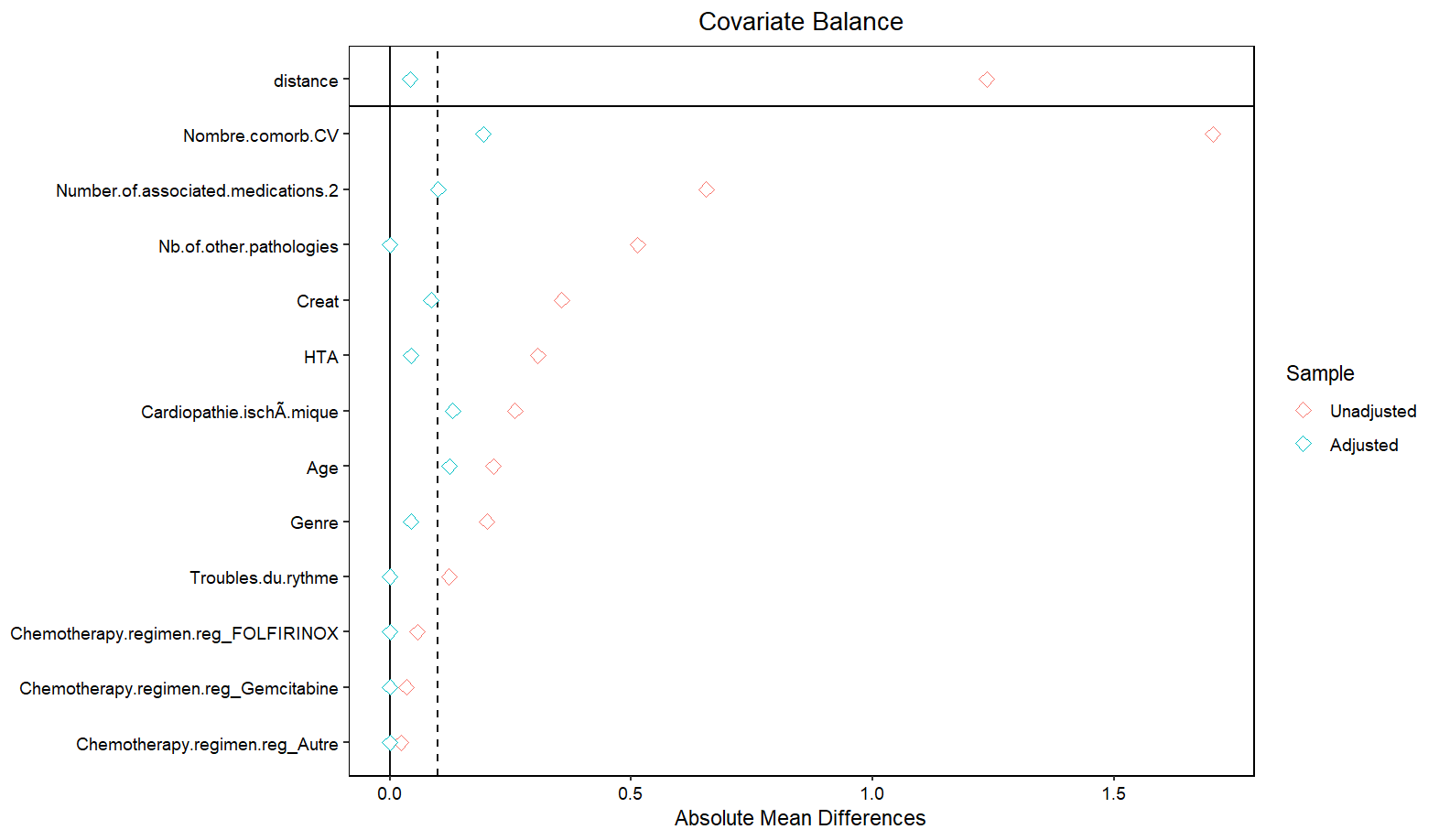
Supplementary Fig. S1: Distribution of the estimated Propensity Score for receiving bêta-blocker

Supplementary Fig. S2: Standardized mean differences in the unmatched and matched sample


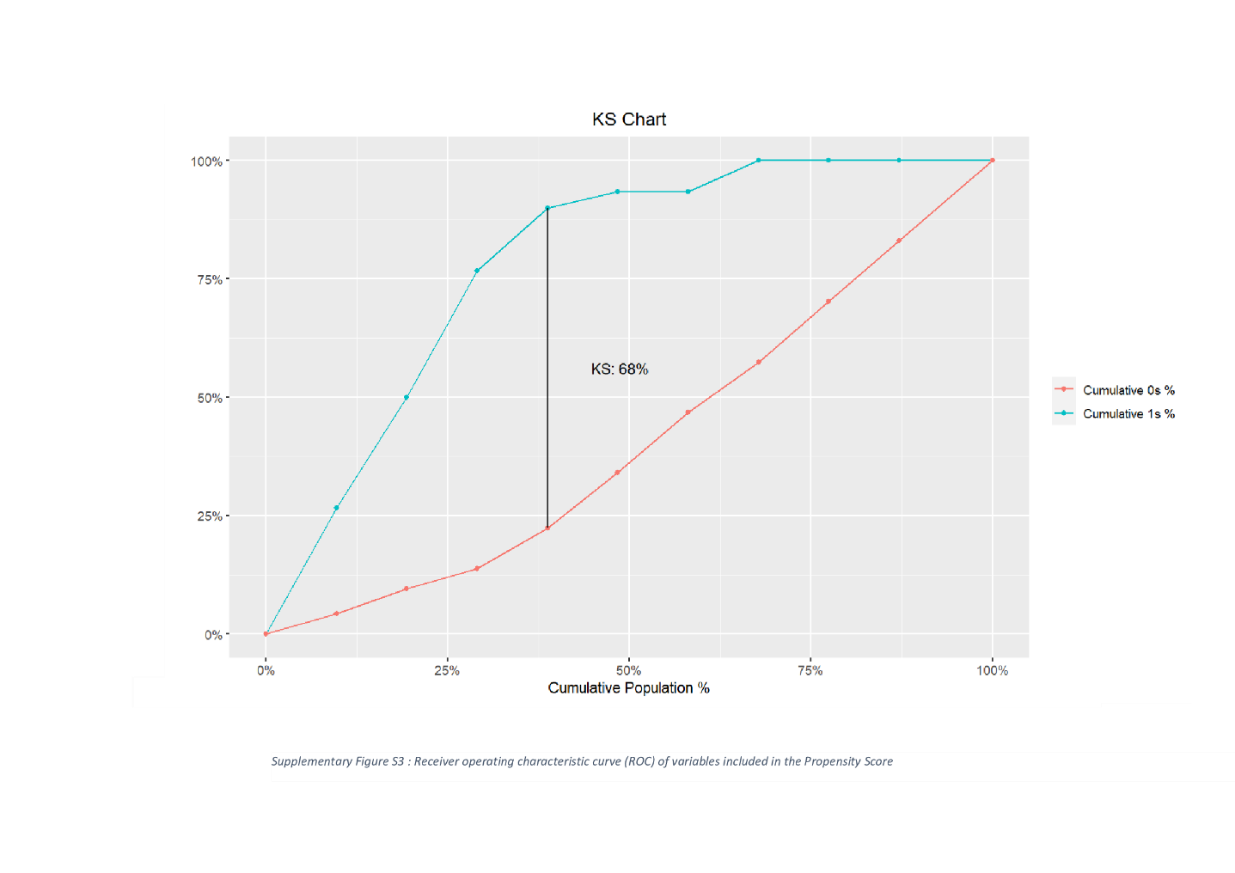


Supplementary Fig. S3: Receiver operating characteristic curve (ROC) of variables included in the Propensity Score

Supplementary Table S1: Univariate analysis according to the event (Overall Survival)

| **Variable** | **Coding (n)** | **Hazard ratios [95% Confidence interval]** | ***p*-value** |
| --- | --- | --- | --- |
| **BB exposure** | No (141)  Yes (41) | **1.48** [0.98-2.23] | 0.06 |
| **BB exposure (time-dependent variable)** | - | **1.87** [0.63-5.54] | 0.30 |
| **Age** | Continous (182) | **1.01** [0.99-1.02] | 0.5 |
| **Age** | Categorial  0 : 18 – 59 yr. (51)  1 : 60 – 69 yr. (54)  2 : ≥ 70 yr. (77) | **ref**  **0.80** [0.52-1.23]  **0.93** [0.63-1.39] | 0.30  0.73 |
| **Sex** | 0 : Women (82)  1 : Men (100) | **1.59** [1.13-2.23] | 0.009 |
| **BMI (kg/m²)** | Continous (182) | **1.01**[0.97-1.04] | 0.8 |
| **BMI (kg/m²)** | Categorial  0 : 18 – 25 kg/m² (104)  1 : 25 – 30 kg/m² (58)  2 : >30 kg/m² (19) | **ref**  **0.87** [0.60-1.26]  **0.92** [0.51-1.65] | 0.46  0.77 |
| **eGFR (mL/min/1.73m²)** | Categorial  0: Stade I (140)  1: Stade II-IIIa (39) | **ref**  **1.05** [0.68-1.62] | 0.82 |
| **Anticancer regimen 1** | 0 : FOLFIRINOX (105)  1 : Gemcitabine (37)  2 : Other (40) | **ref**  **1.48** [0.96-2.30]  **0.53** [0.34-0.82] | 0.08  <10^-2^ |
| **Anticancer regimen 2** | 0 : Chemotherapy with no fluorouracil (40)  1 : Chemotherapy with fluorouracil (142) | **Ref**  **0.60** [0.40-0.90] | 0.01 |
| **Polypharmacy** | 0 : No polypharmacy (57)  1 : 5 - 10 medications (78)  2 : ≥10 medications (47) | **ref**  **1.27** [0.86-1.87]  **1.76** [1.12-2.78] | 0.23  0.01 |
| **Multimorbidity** | 0 : > 5 comorbidities (120)  1 : ≥5 comorbidities (62) | **ref**  **2.17** [1.50-3.16] | <10^-4^ |
| **Cardiovascular condition** | 0 : No (88)  1 : Yes (92) | **ref**  **1.24** [0.88-1.74] | 0.21 |
| **AHT** | 0 : No (102)  1 : Yes (80) | **ref**  **1.22** [0.87-1.72] | 0.26 |
| **MI** | 0 : No (162)  1 : Yes (20) | **ref**  **1.27** [0.75-2.14] | 0.3 |
| **CA** | 0 : No (169)  1 : Yes (13) | **ref**  **3.05** [1.62-5.73] | 0.003 |
| **HF** | 0 : No (180)  1 : Yes (2) | **ref**  **0.31** [0.043-2.23] | 0.2 |
| **Cumulated cardiovascular conditions** | 0: 0 (88)  1: 1 (72)  2: ≥2 (22) | **ref**  **1.18** [0.83-1.70]  **1.51** [0.88-2.57] | 0.36  0.13 |

BB: beta-Blocker; BMI: Body Mass Index; eGFR: estimated Glomerular Filtration Rate; FOLFIRINOX: irinotecan, oxaliplatin, leucovorin, 5-FU; AHT: Arterial Hypertension; CA: Cardiac Arrythmia; HF: Heart failure; MI: Myocardial Ischemia

Supplementary Table S2: Multivariate analysis according to the event (Overall Survival)

| **Variable** | **Coding (n)** | **Hazard ratios [95% Confidence interval]** | | ***p*-value** |
| --- | --- | --- | --- | --- |
| **BB exposure** | No (141)  Yes (41) | **ref**  **1.38** [0.80-2.39] | | 0.25 |
| **BB exposure (time-dependent variable)** | - | **1.02** [0.10-1.45] | | 0.98 |
| **Age** | Categorial  0 : 18 – 59 yr. (51)  1 : 60 – 69 yr. (54)  2 : ≥ 70 yr. (77) | **ref**  **0.63** [0.40-0.99]  **0.66** [0.43-1.04] | | 0.04  0.07 |
| **Sex** | 0 : Women (82)  1 : Men (100) | **1.70** [1.16-2.50] | | <10^-2^ |
| **Anticancer regimen** | 0 : FOLFIRINOX (105)  1 : Gemcitabine (37)  2 : Other (40) | **ref**  **1.69** [1.04-2.74]  **0.58** [0.37-0.93] | | 0.02  0.03 |
| **Polypharmacy** | 0 : No polypharmacy (57)  1 : 5 - 10 medications (78)  2 : ≥10 medications (47) | **ref**  **1.14** [0.75-1.72]  **1.21** [0.67-2.16] | | 0.55  0.53 |
| **Multimorbidity** | 0 : > 5 comorbidities (120)  1 : ≥5 comorbidities (62) | **ref**  **2.14** [1.32-3.45] | | <10^-2^ |
| **Cardiovascular condition** | 0 : No (88)  1 : Yes (92) | **ref**  **0.68** [0.30-1.55] | 0.35 | |
| **AHT** | 0 : No (102)  1 : Yes (80) | **ref**  **1.10** [0.51-2.38] | 0.80 | |
| **MI** | 0 : No (162)  1 : Yes (20) | **ref**  **0.84** [0.42-1.67] | 0.61 | |
| **CA** | 0 : No (169)  1 : Yes (13) | **ref**  **3.53** [1.48-8.38] | 0.004 | |

Supplementary Table S3: Univariate analysis according to the event (Progression-Free Survival)

| **Variable** | **Coding (n)** | **Hazard Ratio [95% Confidence Intervals]** | ***p*-value** |
| --- | --- | --- | --- |
| **BB exposure** | No (141)  Yes (41) | **1.00** [0.60-1.68] | 0.98 |
| **BB exposure (time-dependent variable)** | - | **0.59** [0.19-1.86] | 0.37 |
| **Age** | Continous (182) | **1.00** [0.98-1.02] | 0.65 |
| **Age** | Categorial  0 : 18 – 59 yr. (51)  1 : 60 – 69 yr. (54)  2 : ≥ 70 yr. (77) | **ref**  **0.81** [0.50-1.31]  **0.91** [0.57-1.44] | 0.39  0.68 |
| **Sex** | 0 : Women (82)  1 : Men (100) | **1.09** [0.74-1.61] | 0.67 |
| **BMI (kg/m²)** | Continous (182) | **1.02** [0.98-1.07] | 0.41 |
| **BMI (kg/m²)** | Categorial  0 : 18 – 25 kg/m² (104)  1 : 25 – 30 kg/m² (58)  2 : >30 kg/m² (19) | **ref**  **0.82** [0.53-1.27]  **1.49** [0.80-2.78] | 0.37  0.21 |
| **eGFR (mL/min/1.73m²)** | Categorial  0: Stade I (140)  1: Stade II-IIIa (39) | **ref**  **0.84** [0.50-1.41] | 0.51 |
| **Anticancer regimen 1** | 0 : FOLFIRINOX (105)  1 : Gemcitabine (37)  2 : Other (40) | **ref**  **0.89** [0.48-1.21]  **0.76** [0.47-1.65] | 0.70  0.25 |
| **Anticancer regimen 2** | 0 : Chemotherapy with no fluorouracil (40)  1 : Chemotherapy with fluorouracil (142) | **Ref**  **1.03** [0.58-1.83] | 0.92 |
| **Polypharmacy** | 0 : No polypharmacy (57)  1 : 5 - 10 medications (78)  2 : ≥10 medications (47) | **ref**  **1.21** [0.78-1.86]  **1.15** [0.65-2.03] | 0.40  0.63 |
| **Multimorbidity** | 0 : > 5 comorbidities (120)  1 : ≥5 comorbidities (62) | **ref**  **1.36** [0.84-2.19] | 0.21 |
| **Cardiovascular condition** | 0 : No (88)  1 : Yes (92) | **ref**  **1.32** [0.89-1.97] | 0.36 |
| **AHT** | 0 : No (102)  1 : Yes (80) | **ref**  **1.54** [0.87-1.72] | 0.04 |
| **MI** | 0 : No (162)  1 : Yes (20) | **ref**  **0.88** [0.44-1.75] | 0.72 |
| **CA** | 0 : No (169)  1 : Yes (13) | **ref**  **0.98** [0.31-3.11] | 0.98 |
| **HF** | 0 : No (180)  1 : Yes (2) | **ref**  **1.12** [0.28-4.58] | 0.9 |
| **Cumulated cardiovascular comorbidities** | 0: 0 (90)  1: 1 (72)  2: ≥2 (20) | **ref**  **1.42** [0.94-2.15]  **1.04** [0.51-2.12] | 0.10  0.92 |

BB: beta-Blocker; BMI: Body Mass Index; eGFR: estimated Glomerular Filtration Rate; FOLFIRINOX: irinotecan, oxaliplatin, leucovorin, 5-FU; AHT: Arterial Hypertension; CA: Cardiac Arrythmia; HF: Heart failure; MI: Myocardial Ischemia

Supplementary Table S4: Multivariate analysis according to the event (Progression-Free Survival)

| **Variable** | **Coding (n)** | **HR et IC** | | **pvalue** |
| --- | --- | --- | --- | --- |
| ***BB exposure*** | No (141)  Yes (41) | **ref**  **0.98** [0.50-1.92] | | 0.96 |
| ***BB exposure (time-dependent variable)*** | - | **0.27** [0.065-1.12] | | 0.07 |
| **Anticancer regimen** | 0 : FOLFIRINOX (105)  1 : Gemcitabine (37)  2 : Other (40) | **ref**  **0.71** [0.36-1.37]  **0.76** [0.47-1.21] | | 0.31  0.25 |
| **Multimorbidity** | 0 : > 5 comorbidities (120)  1 : ≥5 comorbidities (62) | **ref**  **1.13** [0.65-1.94] | | 0.67 |
| **Cumulated cardiovascular condition** | 0: 0 (88)  1: 1 (72)  2: ≥2 (22) | **ref**  **0.89** [0.34-2.34]  **0.55** [0.16-1.86] | 0.82  0.34 | |
| **AHT** | 0 : No (102)  1 : Yes (80) | **ref**  **1.88** [0.75-4.72] | 0.18 | |

Supplementary Table S5: Associations between beta-blocker use and progression in the crude analysis, multivariable analysis, and propensity score analyses

| **Parameters** | **Results** | **P-value**** |
| --- | --- | --- |
| ***Number of events/number of patients at risk (%)*** |  | |
| Beta-blocker user (BB+) (n=41) | 18 (43.9%) | - |
| BB- (n=141) | 86 (61.0%) | - |
| ***Crude analysis – HR (95% IC) **** | 1.00 [0.60-1.68] | 0.99 |
| ***Multivariable analysis^‡^*** | 0.95 [0.48-1.88] | 0.88 |
| ***Propensity-score analysis (PS)*** |  | |
| Adjusted for PS^§^ | 1.01 [0.51-2.01] | 0.97 |
| With matching^†^ | 0.73 [0.34-1.56] | 0.42 |
| With inverse probability-weighting ^φ^ | 0.96 [0.54-1.70] | 0.90 |

* All variables integrated in Cox regression models validated proportional hazard and log-linearity assumptions (for continuous variables)

**Likelihood ratio test

‡ Hazard ratio from the multivariable Cox proportional-hazards model, with adjustment for anticancer regimen, multimorbidity, presence of cardiovascular comorbidity and AHT. The analysis included 94 patients.

§ Hazard ratio from a multivariable Cox proportional-hazards model with additional adjustment for the propensity score. The analysis included 182 patients.

† Hazard ratio from a multivariable Cox proportional-hazards model with matching according to the propensity score. The analysis included patients 60 patients (30 who received BB and 30 who did not) according to the nearest method with a caliper fixed at 0.2.

φ Hazard ratio from the multivariate Cox proportional-hazards model with inverse probability weighting according to the propensity score. The analyses included 182 patients.
